# Association of a novel seven-gene expression signature with the disease prognosis in colon cancer patients

**DOI:** 10.18632/aging.102365

**Published:** 2019-10-15

**Authors:** Haojie Yang, Hua Liu, Hong-Cheng Lin, Dan Gan, Wei Jin, Can Cui, Yixin Yan, Yiming Qian, Changpeng Han, Zhenyi Wang

**Affiliations:** 1 Department of Coloproctology, Yueyang Hospital of Integrated Traditional Chinese and Western Medicine, Shanghai University of Traditional Chinese Medicine, Shanghai 200437, China; 2 Department of Coloproctology, The Sixth Affiliated Hospital of Sun Yat-sen University, Gastrointestinal and Anal Hospital of Sun Yat-sen University, Guangzhou 510655, China; 3 Department of Emergency Medicine, Yueyang Hospital of Integrated Traditional Chinese and Western Medicine, Shanghai University of Traditional Chinese Medicine, Shanghai 200437, China

**Keywords:** colon cancer, gene expression profile, signature, ceRNA

## Abstract

Older patients who are diagnosed with colon cancer face unique challenges, specifically regarding to cancer treatment. The aim of this study was to identify prognostic signatures to predicting prognosis in colon cancer patients through a detailed transcriptomic analysis. RNA-seq expression profile, miRNA expression profile, and clinical phenotype information of all the samples of TCGA colon adenocarcinoma were downloaded and differentially expressed mRNAs (DEMs), differentially expressed lncRNAs (DELs) and differentially expressed miRNAs (DEMis) were identified. A competing endogenous RNA (ceRNA) network was constructed further and DEMs related with prognosis in the ceRNA network was screened using Cox regression analysis. Risk score models for predicting the prognosis of colon cancer patients were built using these DEMs. A total of 1476 DEMs, 9 DELs, and 243 DEMis between the tumor and normal samples were identified and functional enrichment analyses showed that the DEMs were significantly enriched in the nervous system development, ribosome biogenesis pathways in eukaryotes, and drug metabolism cytochrome P450. Twelve DEMs related with prognosis were screened from the ceRNA network. Thereafter, the risk score models of prognostic DEMs were obtained, involving seven DEMs (*SGCG, CLDN23, SLC4A4, CCDC78, SLC17A7, OTOP3,* and *SMPDL3A*). Additionally, cancer stage was identified as a prognostic clinical factor. This prognostic signature was further validated in two independent datasets. Our study developed a seven-mRNA and one-clinical factor signature that are associated with prognosis in colon cancer patients, which may serve as possible biomarkers and therapeutic targets in the future.

## INTRODUCTION

Colon cancer is the third most common cancer, with increasing morbidity and mortality worldwide [1]. The morbidity of colon cancer increases along with the increase of age. In fact, more than 60% of people who developed cancer are order than 65. Despite the significant progress in tailored therapy, the high migration and invasion capabilities of this tumor have been a bottleneck in reducing the mortality, keeping its 5-year survival rate under 12% [2, 3]. It has been reported that the prognosis of colon cancer mainly depends on the clinicopathological features and the tumor stage. Nevertheless, due to the remarkable disease heterogeneity, it is difficult to determine the prognosis of patients based on these traditional factors [4]. Therefore, a better understanding of the pathogenesis and identification of new promising prognostic biomarkers are essential for development of effective therapies for colon cancer patients.

A recent study has reported that the identification of prognostic signature based on the genomic or transcriptome data can improve our understanding of cancer progression and survival rate [5]. Presently, many prognostic gene-expression signatures have been identified in various human cancers which help in predicting the cancer prognosis outcomes [6–8]. Among the various gene-expression signatures in colon cancer, the six-cluster gene expression ColoPrint [9] and Colon OncotypeDx [7] have been widely verified in retrospective studies. Recently, a study has reported that the protein-coding genes make up less than 2% of the whole genome sequences, and the noncoding genes are transcribed into noncoding RNAs, such as microRNAs (miRNAs) and long noncoding RNAs (lncRNAs) [10]. Some of the recent studies have demonstrated the competing endogenous RNA (ceRNA) activity of lncRNAs, as natural miRNA decoys in human pathophysiological conditions, including cancer [11]. Therefore, systematic analysis of lncRNA-associated ceRNA network may help to explore and identify more prognostic gene-expression signatures in colon cancer.

In this study, we aimed to screen newer prognostic signatures for colon cancer through the analysis of RNA-seq expression profile and miRNA expression profiles. Based on the differentially expressed mRNAs (DEMs), lncRNA (DELs), and miRNA (DEMis) data obtained between the tumor and normal samples, a ceRNA network was constructed and DEMs involved in this network were selected for the construction of prognostic risk model. Our study presented some novel biomarkers with a potential prognostic value, and provided a preliminary bioinformatic evidence for understanding the complex mechanism of colon cancer progression.

## RESULTS

### Differential analysis

**Figure 1:**
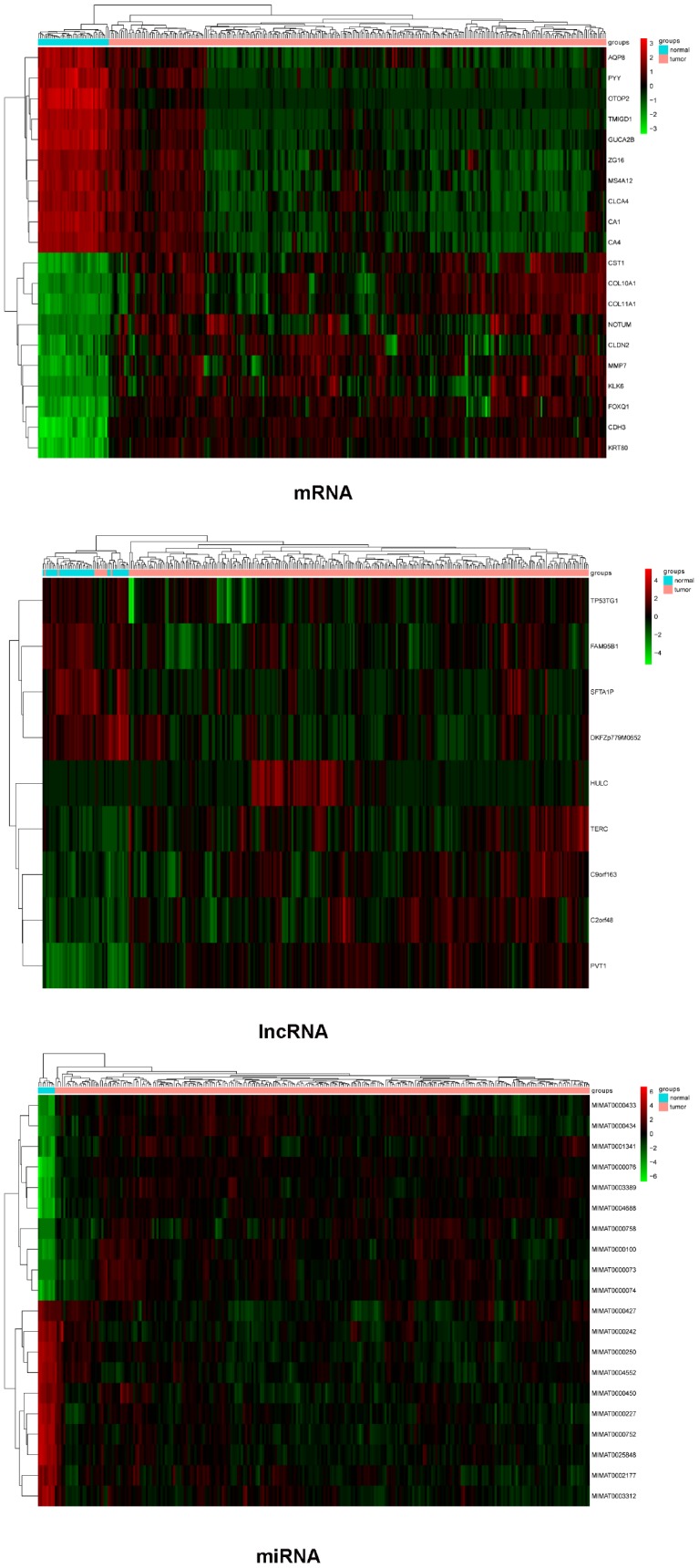
**Heatmaps of differential expression mRNAs (top 10), lncRNAs, and miRNAs (top 10).** Red represents high expression and green represents low expression.

The mRNA, lncRNA, and miRNA expression profiles obtained were subjected to differential expression analysis. The screening thresholds for mRNA and miRNA were adjusted *P* value < 0.01 and |logFC| > 2, and for lncRNA was adjusted *P* value < 0.05 and |logFC| > 1. A total of 1476 (323 upregulated and 1153 downregulated) DEMs, 9 (5 upregulated and 4 downregulated) DELs, and 243 (113 upregulated and 130 downregulated) DEMis were identified. Top10 genes (in ascending order according to the adjusted *P* value) were selected from DEMs, DELs, and DEMis to draw the heatmaps, as shown in Figure 1.

### GO enrichment analysis of DEMs

**Figure 2:**
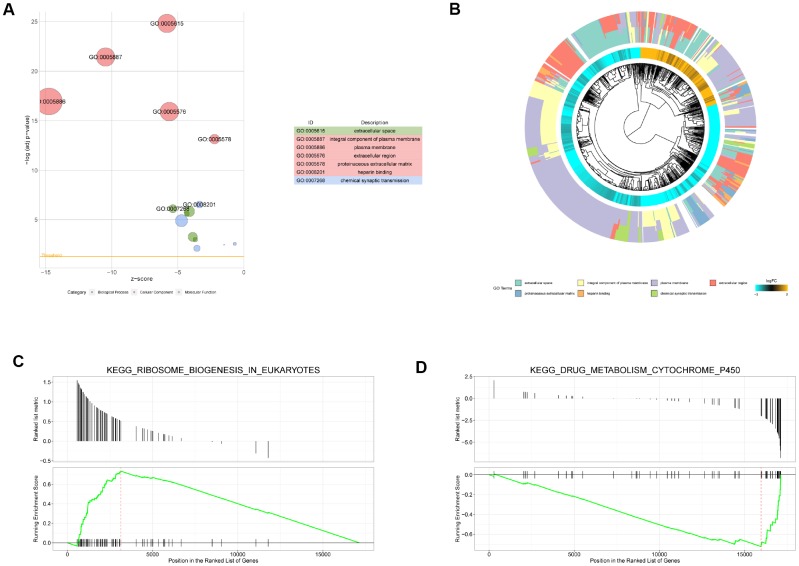
**Gene Ontology (GO) and gene set enrichment analysis (GSEA) enrichment analysis.** (**A**) Bubble diagram of GO enrichment analysis (top 7). The circle size indicates the number of enriched genes. Green represents biological process, red represents molecular function, and blue represents cellular component. (**B**) Cluster diagram of GO enrichment analysis. (**C**) The upregulated pathway (KEGG_RIBOSOME_BIOGENESIS_IN_EUKARYOTES) of GSEA analysis with the highest enrichment score. (**D**) The downregulated pathway (KEGG_DRUG_METABOLISM_CYTOCHROME_P450) with the highest enrichment score.

With a threshold of adjusted *P* value < 0.01, 13 BPs, 14 CCs, and 5 MFs were significantly enriched by the DEMs. The top 5 terms for the three categories are shown in Figure 2A. The seven most significant terms are shown in detail. According to the genes in term, the clustering of genes was carried out to construct the phylogenetic tree (Figure 2B). In terms of the involved BP terms, the DEMs were significantly enriched in chemical synaptic transmission, axon guidance, nervous system development, cell adhesion, and transport. From the perspective of CC terms, the encoding proteins were mainly located in an integral component of the plasma membrane, plasma membrane, and apical plasma membrane.

### Pathway enrichment analysis

According to the KEGG pathway enrichment analysis of the DEMs obtained above by GSEA, a total of 67 significantly enriched pathways were identified (adjusted *P* value < 0.05), among which 16 were upregulated pathways (normalized enrichment score (NES) > 0) and 51 were downregulated pathways (NES < 0). KEGG ribosome biogenesis in eukaryotes had the largest NES in the significantly upregulated pathways (Figure 2C). In addition, DNA replication, RNA degradation, IL17 signaling pathway, p53 signaling pathway, and oxytocin signaling pathway were also significantly upregulated. Among the significantly downregulated pathways, drug metabolism cytochrome P450 had the largest NES (Figure 2D). Additionally, chemical carcinogenesis and mineral absorption pathways were also downregulated.

### Co-expression analysis

**Figure 3:**
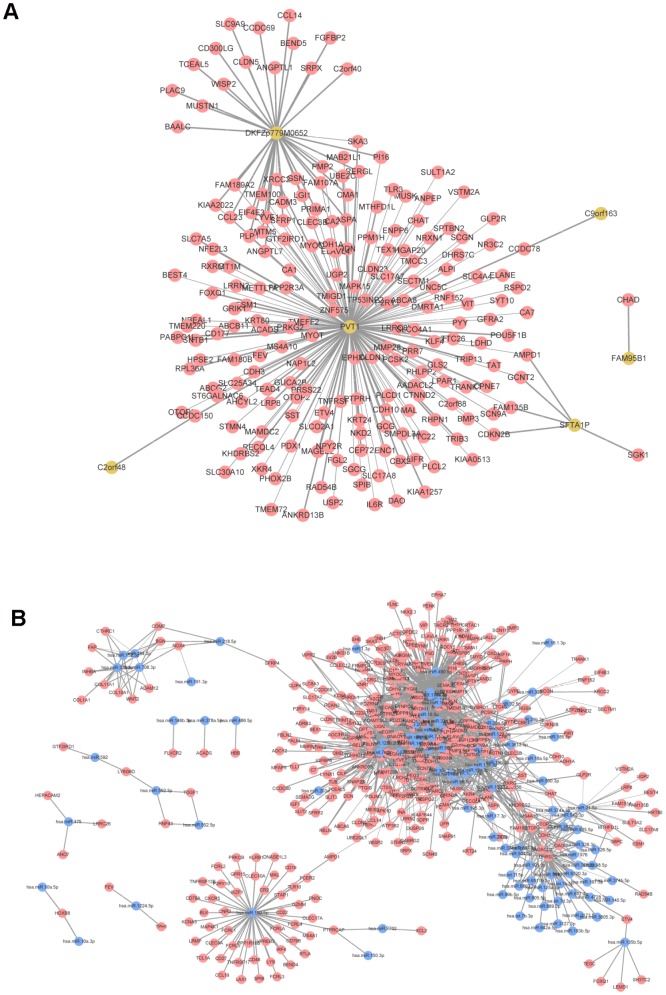
**Co-expression network analysis.** (**A**) mRNA-lncRNA co-expression network. (**B**) mRNA-miRNA co-expression network. Red node was mRNA, yellow node was lncRNA, and blue node was miRNA. The line thickness indicates the relative size of correlation coefficient.

For the screened DELs and DEMis, the correlation coefficients and statistical significance degree between them and DEMs were calculated, respectively, and the gene pairs with |r| > 0.6 and an adjusted *P* value < 0.01 were screened out. There were 193 nodes (199 mRNAs and 6 lncRNAs) and 229 edges in the co-expression network of mRNA-lncRNA, where lncRNA Pvt1 oncogene (PVT1) had the highest connectivity degree (Figure 3A). In the co-expression network of mRNA-miRNA, there were 184 nodes (392 mRNAs, 102 miRNAs) and 1,935 edges (Figure 3B).

### miRNA prediction

**Figure 4:**
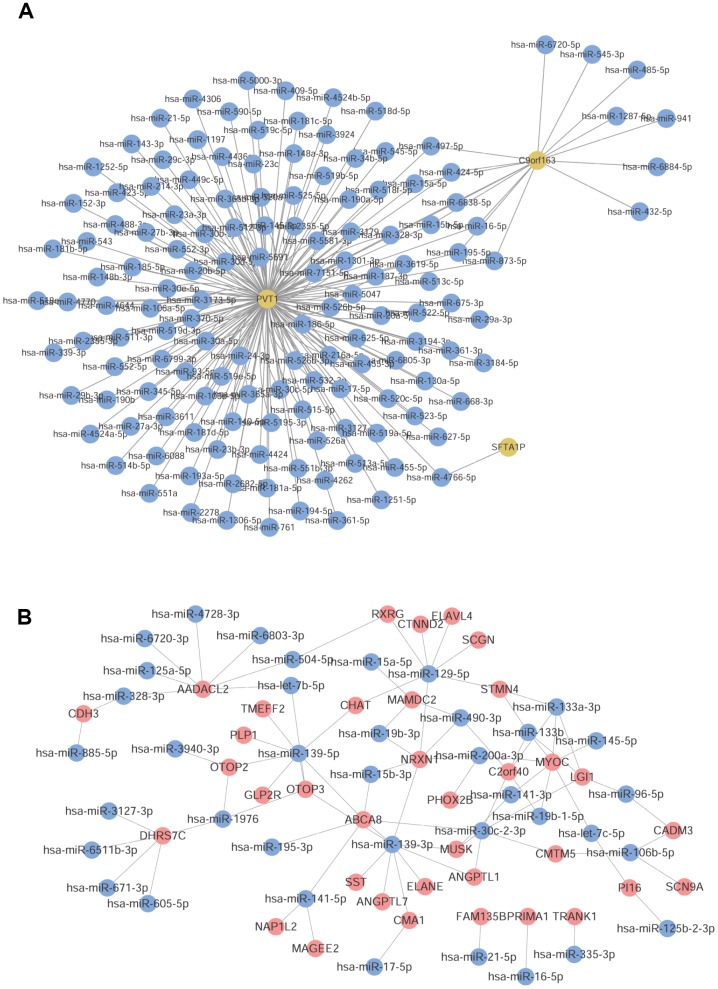
**Prediction of common miRNAs.** (**A**) LncRNA-miRNA pairs predicted by lncRNA. (**B**) The obtained common mRNA-miRNA. Red node was mRNA, yellow node was lncRNA, and blue node was miRNA.

A total of 200 lncRNA-miRNA pairs (3 lncRNAs and 141 miRNAs) were predicted, based on the lncRNA involved in lncRNA-mRNA co-expression pairs using starbase (Figure 4A). Additionally, the mRNA involved in lncRNA-mRNA co-expression pairs were subjected to mRNA-miRNA prediction, and 117,570 pairs of miRNA-mRNA (199 mRNAs and 2498 miRNAs) were obtained with Score = 1 and Position = CDS. These mRNA-miRNA pairs were intersected with the obtained mRNA-miRNA co-expression pairs, and 89 mRNA-miRNA pairs (36 mRNAs and 38 miRNAs) were further screened (Figure 4B).

### LncRNA and miRNA pathway enrichment analyses

DKFZp779M0652 and PVT1 that had many target genes were subjected to functional enrichment. DKFZp779M0652 was associated with functions of cellular response to tumor necrosis factor and cellular response to interleukin-1. PVT1 was related to one-carbon metabolic process and reciprocal meiotic recombination.

The mRNAs in the miRNA-mRNA relation pairs obtained above were used as the target genes of miRNAs. Since there were few target genes and enrichment analysis could not be carried out, thus the functions of each target gene were inquired on the genecards to obtain the functions of all miRNA (data not shown).

### ceRNA network construction

**Figure 5:**
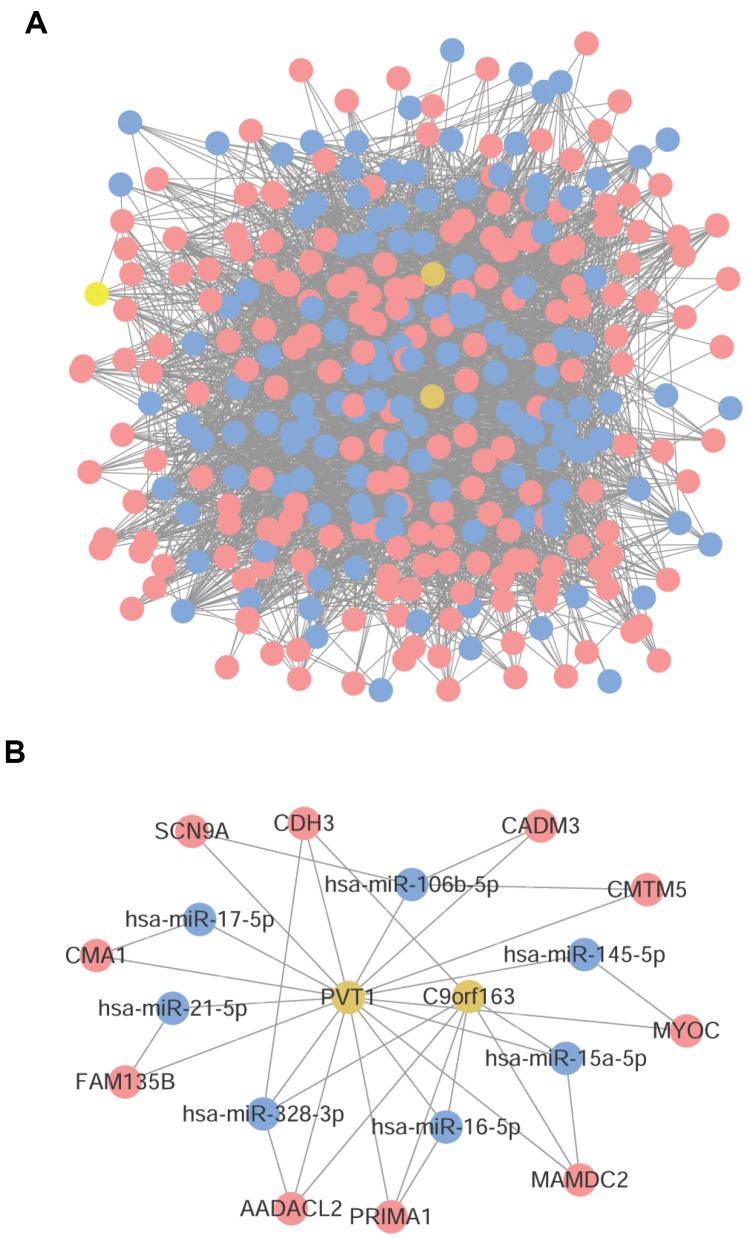
**Construction of ceRNA network.** (**A**) The ceRNA network constructed by 197 mRNAs, 135 miRNAs, and 3 lncRNAs. (**B**) The ceRNA network constructed by 7 miRNAs, 2 lncRNAs, and 10 mRNAs. Red node was mRNA, yellow node was lncRNA, and blue node was miRNA.

A total of 8,353 lncRNA-miRNA-mRNA pairs were obtained, involving 197 mRNAs, 135 miRNAs, and 3 lncRNAs. The constructed ceRNA network is shown in Figure 5A. After further screening of the network, only the aforementioned co-expressed mRNA-miRNA pairs were retained in the mRNAs-miRNA relation pairs. Finally, a total of 21 lncRNA-miRNA-mRNA pairs were obtained, including 7 miRNAs, 2 lncRNAs, and 10 mRNAs. The network is depicted in Figure 5B.

### Establishment of mRNA prognostic risk model

**Figure 6:**
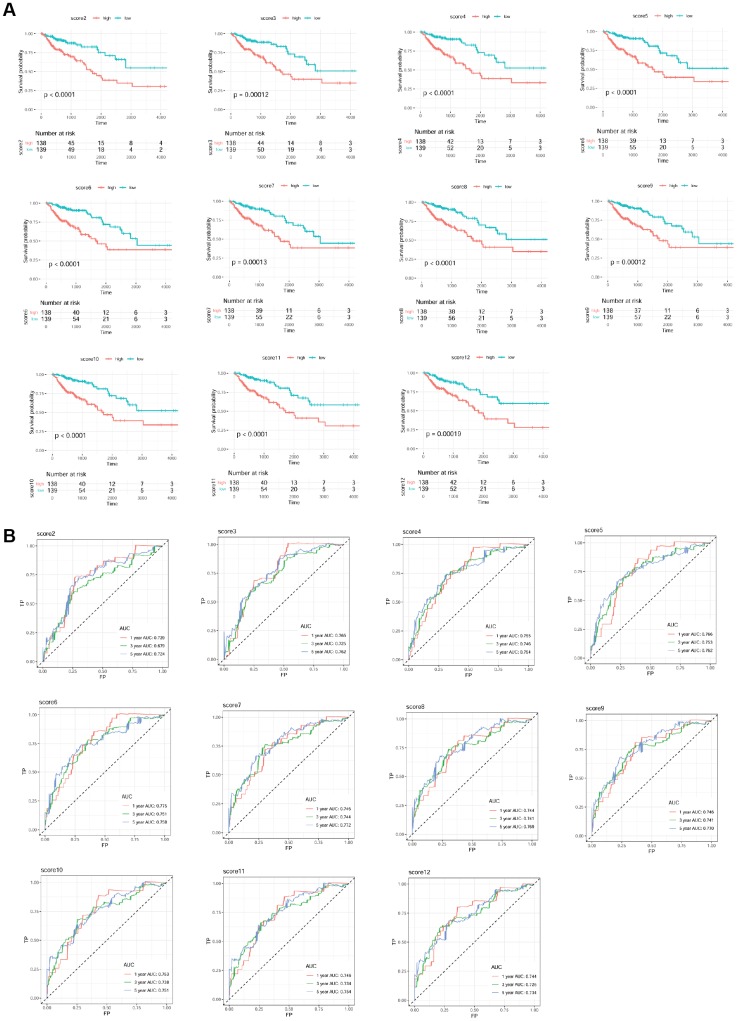
Survival curve (**A**) and receiver operation characteristic curve (**B**) of each scoring model.

A total of 277 samples (68 deceased and 209 living) with clinical information of overall survival time were selected. 197 mRNAs from ceRNA network were used as candidate mRNAs. The expression level of mRNA in the sample set was screened, and univariable Cox regression analysis was conducted based on the clinical prognosis information of the sample. The regression threshold of *P* value was set at 0.05 to screen the prognostic mRNAs, as shown in Table 1. The risk score models of prognostic mRNAs are shown in Table 2. The drawn survival curves are shown in Figure 6A. All models were significantly correlated, and the ROC curves were drawn based on the risk scores of all the models and survival information (Figure 6B). AUC values were calculated (Table 3), and the time periods were 1, 3, and 5 years, respectively. The largest AUC of 5-year prognosis scoring model was used as the final risk score model: Risk score = 0.58343 * exp (*SGCG*) + (-0.11860) * exp (*CLDN23*) + (-0.09726) * exp (*SLC4A4*) + 0.18416 * exp (*CCDC78*) + 0.13586 * exp (*SLC17A7*) + 0.40269 * exp (*OTOP3*) + (-0.23459) * exp (*SMPDL3A*). The parameters of mRNAs (*SGCG*, *CLDN23*, *SLC4A4*, *CCDC78*, *SLC17A7*, *OTOP3,* and *SMPDL3A*) associated with the optimal prognosis are shown in Table 4.

**Table 1:** Prognostic mRNAs obtained from univariate Cox regression.

**mRNA**	**HR (95% CI for HR)**	**Beta**	**P value**
SGCG	1.8 (1.3-2.4)	0.57	0.00019
CLDN23	0.73 (0.57-0.92)	-0.32	0.0078
SLC4A4	0.89 (0.81-0.97)	-0.12	0.0085
CCDC78	1.2 (1-1.4)	0.2	0.011
SLC17A7	1.4 (1.1-1.8)	0.33	0.011
OTOP3	1.4 (1-2)	0.36	0.027
SMPDL3A	0.74 (0.56-0.97)	-0.3	0.029
TCEAL5	1.3 (1-1.6)	0.23	0.033
MAB21L1	1.2 (1-1.5)	0.22	0.033
CDH10	1.7 (1-2.6)	0.5	0.036
CA2	0.88 (0.78-0.99)	-0.13	0.036
SLC17A8	0.67 (0.46-0.98)	-0.4	0.037

**Table 2:** Risk score model.

**Score number**	**Risk score model**
Score1	0.5700*exp(SGCG)
Score2	0.5700*exp(SGCG)+(-0.3194)*exp(CLDN23)
Score3	0.57915*exp(SGCG)+(-0.23856)*exp(CLDN23)+(-0.10073)*exp(SLC4A4)
Score4	0.66751*exp(SGCG)+(-0.16306)*exp(CLDN23)+(-0.09054)*exp(SLC4A4) +0.19449*exp(CCDC78)
Score5	0.56759*exp(SGCG)+(-0.15242)*exp(CLDN23)+(-0.10090)*exp(SLC4A4) +0.18517*exp(CCDC78)+0.18698*exp(SLC17A7)
Score6	0.57012*exp(SGCG)+(-0.13823)*exp(CLDN23)+(-0.11492)*exp(SLC4A4) +0.18897*exp(CCDC78)+0.13280*exp(SLC17A7)+0.39265*exp(OTOP3)
Score7	0.58343*exp(SGCG)+(-0.11860)*exp(CLDN23)+(-0.09726)*exp(SLC4A4) +0.18416*exp(CCDC78)+0.13586*exp(SLC17A7)+0.40269*exp(OTOP3)+(-0.23459)*exp(SMPDL3A)
Score8	0.53201*exp(SGCG)+(-0.13415)*exp(CLDN23)+(-0.10341)*exp(SLC4A4) +0.18600*exp(CCDC78)+0.09211*exp(SLC17A7)+0.39503*exp(OTOP3)+(-0.22168)*exp(SMPDL3A)+0.10713*exp(TCEAL5)
Score9	0.52245*exp(SGCG)+(-0.13418)*exp(CLDN23)+(-0.10215)*exp(SLC4A4) +0.18929*exp(CCDC78)+0.08428*exp(SLC17A7)+0.39404*exp(OTOP3)+(-0.22316)*exp(SMPDL3A)+0.09120*exp(TCEAL5)+0.03316*exp(MAB21L1)
Score10	0.50527*exp(SGCG)+(-0.14585)*exp(CLDN23)+(-0.10778)*exp(SLC4A4) +0.19778*exp(CCDC78)+0.06656*exp(SLC17A7)+0.40789*exp(OTOP3)+(-0.21657)*exp(SMPDL3A)+0.04899*exp(TCEAL5)+(-0.02491)*exp(MAB21L1)+0.41369*exp(CDH10)
Score11	0.51080*exp(SGCG)+(-0.14221)*exp(CLDN23)+(-0.09733)*exp(SLC4A4) +0.19483*exp(CCDC78)+0.07246*exp(SLC17A7)+0.41640*exp(OTOP3)+(-0.20729)*exp(SMPDL3A)+0.03954*exp(TCEAL5)+(-0.02730)*exp(MAB21L1)+0.41328*exp(CDH10)+(-0.02849)*exp(CA2)
Score12	0.46042*exp(SGCG)+(-0.08915)*exp(CLDN23)+(-0.09846)*exp(SLC4A4) +0.19869*exp(CCDC78)+0.08603*exp(SLC17A7)+0.50655*exp(OTOP3)+(-0.16637)*exp(SMPDL3A)+0.02671*exp(TCEAL5)+(-0.05120)*exp(MAB21L1)+0.54879*exp(CDH10)+(0.01309)*exp(CA2)+(-0.36081)*exp(SLC17A8)

**Table 3:** The Area Under The Curve (AUC) of 1 year, 3 year and 5year, with 95% confidence interval (CI).

**Score number**	**1 year (95% CI)**	**3 year (95% CI)**	**5 year (95% CI)**
score2	0.729 (0.641-0.802)	0.679 (0.631-0.751)	0.724 (0.652-0.805)
score3	0.765 (0.715-0.817)	0.725 (0.679-0.790)	0.762 (0.669-0.836)
score4	0.755 (0.690-0.820)	0.746 (0.681-0.815)	0.754 (0.688-0.849)
score5	0.766 (0.726-0.831)	0.753 (0.677-0.825)	0.762 (0.686-0.878)
score6	0.775 (0.733-0.837)	0.751 (0.700-0.809)	0.758 (0.685-0.886)
score7	0.746 (0.695-0.837)	0.744 (0.720-0.808)	0.772 (0.705-0.833)
score8	0.744 (0.689-0.839)	0.741 (0.698-0.809)	0.769 (0.678-0.838)
score9	0.746 (0.690-0.842)	0.741 (0.693-0.812)	0.77 (0.675-0.837)
score10	0.753 (0.712-0.838)	0.738 (0.684-0.800)	0.751 (0.671-0.825)
score11	0.746 (0.705-0.833)	0.734 (0.691-0.792)	0.754 (0.676-0.829)
score12	0.744 (0.701-0.833)	0.726 (0.660-0.808)	0.734 (0.637-0.824)

**Table 4:** The parameters of mRNAs associated with the optimal prognosis

**mRNA**	**Coef**	**Hazard ratio**	**Pr(>|z|)**	**Signif.**
SGCG	0.58343	1.79217	0.00128	**
CLDN23	-0.1186	0.88816	0.37088	
SLC4A4	-0.09726	0.90732	0.07544	.
CCDC78	0.18416	1.2022	0.02445	*
SLC17A7	0.13586	1.14552	0.33808	
OTOP3	0.40269	1.49584	0.02125	*
SMPDL3A	-0.23459	0.7909	0.12177	

Coef is the coefficient value obtained by Cox-PH regression model (positive value means positive correlation with survival time, negative value means negative correlation with survival time); Hazard Ratio is the risk score, and p value is the significance threshold of the test.

### Cox regression analysis of prognostic clinical factors

**Figure 7:**
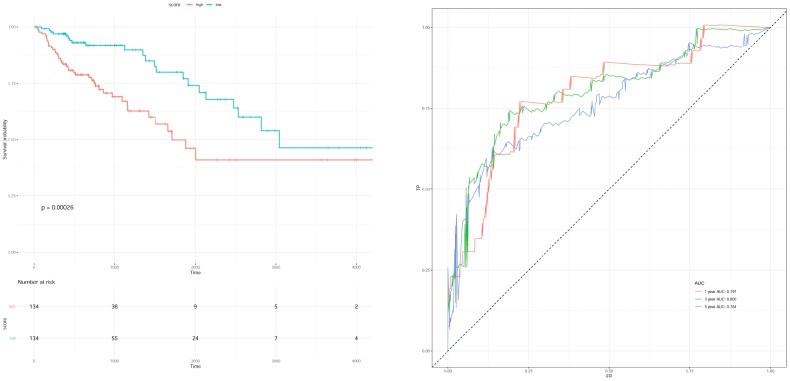
Survival curve (**A**) and receiver operation characteristic curve (**B**) of scoring model with clinical factor of stage.

The clinical factors of gender, age at initial pathologic diagnosis anatomic neoplasm subdivision, and pathologic stage were selected for univariable Cox regression analysis combining with the survival information, and the influence factor of pathologic stage (*P* < 0.0001) was selected. The samples without pathologic stage were removed from 277 samples in the previous step, and the remaining 268 samples were used for subsequent analysis. Pathologic stage contained the following types: “Stage I”, “Stage IA”, “Stage II”, “Stage IIA”, “Stage IIB”, “Stage IIC”, “Stage III”, “Stage IIIA”, “Stage IIIB”, “Stage IIIC”, “Stage IV”, “Stage IVA”, and “Stage IVB”. These types were converted into stage “1”, “2”, “3”, and “4”, and were denoted as “stage n” which was added into the model above for multivariable Cox regression analysis (p = 0.00026) to obtain the new scoring model as follows: Risk score = 0.51396 * exp (*SGCG*) + (-0.16881) * exp (*CLDN23*) + (-0.08028) * exp (*SLC4A4*) + 0.11820 * exp (*CCDC78*) + 0.09786 * exp (*SLC17A7*) + 0.44172 * exp (*OTOP3*) + (-0.22250) * exp (*SMPDL3A*) + 0.66818 * stage_n. The parameters of scoring model are shown in Table 5. The survival curve and ROC curve of survival rate at 1, 3, and 5 years are shown in Figure 7.

**Table 5:** The parameters of scoring model.

**mRNA**	**Coef**	**Hazard ratio**	**Pr(>|z|)**	**Signif.**
SGCG	0.51396	1.67189	0.00706	**
CLDN23	-0.16881	0.84467	0.20849	
SLC4A4	-0.08028	0.92285	0.14911	
CCDC78	0.1182	1.12547	0.14434	
SLC17A7	0.09786	1.10281	0.51499	
OTOP3	0.44172	1.55538	0.00821	**
SMPDL3A	-0.2225	0.80052	0.14536	
stage_n	0.66818	1.95069	1.91E-05	***

### Model validation using independent datasets

**Figure 8:**
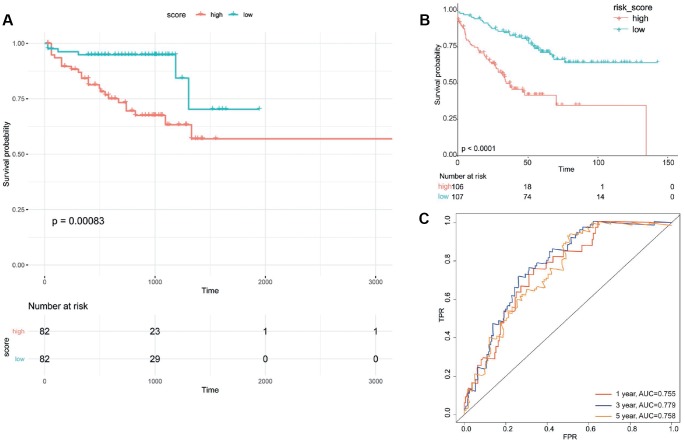
**Validation of obtained scoring model.** Survival curve of patients in high-risk group and low-risk group divided according to prognostic signature for UCSC Xene database (**A**) and GSE17538 (**B**). (**C**) Area under receiver operation characteristic curve (AUC) for 1/3/5 survival rate of patients in GSE17538. FPR, false positive rate; TPR, true positive rate.

Risk score values of all the samples in two independent validation datasets were calculated based on the above risk score model. All samples were divided into high risk group and low risk group according to the median score of risk score. Kaplan-Meier survival curve analysis showed that the developed prognostic signature could well distinguish high-risk and low-risk groups; the survival time of patients in low-risk group was significantly longer than that of patients in high-risk group (*P* = 0.00083 and *P* < 0.0001) (Figure 8A and 8B). The AUCs of this prognostic signature in predicting 1/3/5 survival rate of patients in GSE17538 were 0.755, 0.799 and 0.758, respectively (Figure 8C).

## DISCUSSION

Enhanced migration and invasion potential of the colon cancer cells have led to a significant low 5-year survival rate for the colon cancer patients. Thus, an accurate prediction of prognosis is crucial for the personalized treatment of these patients. Nowadays, gene expression profiling has become a commercial adjunct to cancer therapy. For instance, a six-lncRNA expression signature is utilized as an indicator to evaluate the prognosis of colorectal cancer patients [12]. However, the prognostic tools available for patients with colon cancer usually do not include clinical factors. In this study, we identified a seven mRNAs and one clinical factor-based prognostic signature for colon cancer, which was proved to predict colon cancer reliably.

Among the seven prognosis-related mRNAs, two were from solute carrier family, including solute carrier family 4 member 4 (*SLC4A4*) and *SLC17A7*. The members of solute carrier family have been identified as tumor suppressors. For instance, methylation in the CpG islands of the *SLC25A43* gene has been suggested to be a possible mechanism of gene silencing in breast cancer without loss of heterozygosity [13]. *SLC5A8* is another tumor suppressor gene, which is often downregulated by promoter hypermethylation in pancreatic cancer [14]. Lin et al. [15] recently identified *SLC17A7* as a tumor suppressor gene in glioblastoma, which inhibited cell proliferation and invasion of the cancer cells. *SLC4A4* was found to be significantly downregulated in the clear cell renal cell carcinoma tissues, low expression of which was corelated with poor prognosis [16]. Although their roles in colon cancer have not been clearly elucidated, we speculated that the two solute carrier family genes may server as key prognostic factors of colon cancer pathogenesis.

Claudin 23 (CLDN23) belongs to the claudin family, encoding proteins with four transmembrane domains associated with the formation of tight junctions among adjacent cells [17]. Our study also revealed that *CLDN23* was involved in the pathway of cell adhesion molecules and GO associated with plasma membrane, suggesting that it may play a role in the communication and interaction between cells. Previously, a study had reported that *CLDN23* is downregulated in tumors of colorectal cancer and the downregulated level is correlated with the prognosis of colorectal cancer patients [18].

For the other four genes (*SGCG*, *CCDC78*, *OTOP3,* and *SMPDL3A*) in the predicted scoring model, their roles in human cancers have not yet been fully investigated. Nevertheless, the present results showed that all the seven mRNAs had interactions with PVT1. PVT1 encodes a lncRNA, which maps to chromosome 8q24 (8q24) [19]. It is known that 8q24 amplification is a frequent event in various malignant diseases, including colorectal cancer. For instance, the oncogene MYC has been mapped to 8q24 [20]. Shtivelman and Bishop et al*.* [21] have reported that PVT1 and MYC are co- amplified in colorectal cancer cell lines. A recent study demonstrated that PVT1 can generate antiapoptotic activity in colorectal cancer cells and abnormal expression of this gene could be a prognostic factor in colorectal cancer patients [22]. Yu et al. [23] also suggested that PVT1 functions as an oncogene to promote proliferation and metastasis of colon cancer cells in humans through the miR-30d-5p/RUNX2 axis. Taken together, we speculated that these prognosis-related mRNAs may play roles in colon cancer by interacting with PVT1.

In addition to these gene signatures, our study also identified a clinical factor (stage) associated with the prognosis of colon cancer patients. It is well known that The American Joint Committee on Cancer TNM staging system is currently the gold standard for determining the prognosis of colon cancer patients. The 5-year survival rate for patients with stage I is about 93%, for patients with stage II decreases to 80%, and for patients with stage III is only 60% [24]. Therefore, the identification of clinical prognostic factor of stage in this study further suggested the reliability of our results.

Strength of this study is that we identified a prognostic signature consisting of 7 DEMs and one clinical factor and this prognostic signature could predict the 1/3/5-year survival rate with relatively higher AUC in both training dataset and validation dataset. However, there are some limitations in this study. First, the differential expression of the 7 DEMs were identified from RNA-seq data and lack of experimental validation. Though the RNA-seq data of TCGA are of high quality, further experimental validation is still warranted. Besides, further *in vitro* and *in vivo* investigations of the functions of these 7 DEMs in colon cancer are also needed.

In conclusion, our study reveals a seven-mRNA and one-clinical factor signature that is associated with prognosis in colon cancer patients. This signature may serve as a possible candidate biomarker and therapeutic target for colon cancer patients. Pre-clinical studies followed by clinical trials are needed to validate our findings in the future.

## METHODS

### Public data processing

RNA-seq expression profile, miRNA expression data, and clinical phenotype information of all the samples of TCGA colon adenocarcinoma were downloaded from the University of California Santa Cruz (UCSC) Xene database [16] on May 27^th^, 2019. RNA-seq data from 329 samples, including 288 tumor tissues and 41 normal tissues and miRNA expression data from 261 samples, including 253 tumor tissues and 8 normal tissues were downloaded. Besides, clinical information of 551 patients, including age, gender, tumor histological grading, survival time and survival status, were also downloaded.

### LncRNA/mRNA re-annotation

GENCODE database is a scientific project in genome research which was used to identify and map all protein-coding genes within the ENCODE regions [24]. The gtf gene annotation file (Release 26, GRCh38.p10) provided by GENCODE database was downloaded, and the downloaded RNA-seq expression data were re-annotated with mRNA and lncRNA to obtain the mRNA expression profile and lncRNA expression profile, respectively. The gene with annotation information of "protein coding" was retained as mRNA, and with annotation information of "antisense", "sense intronic", "lincRNA", "sense overlapping", "processed transcript", "3prime overlapping ncRNA", or "non-coding" was retained as lncRNA. The clinical phenotype information corresponding to the gene expression profile of the samples were screened.

### Differential analysis

The log2(count+1) data were transformed into raw count data and normalized into distribution of same mean value and equal variance for each sample using betaqn function in R package. The differences between tumor and normal samples in the three expression profiles were analyzed using the Bayesian method in limma package (version 3.40.0) [12]. Significance test was performed using the paired t-test to obtain the *P* values of all the genes. The *P* values were then adjusted for multiple test using Benjamini & Hochberg (BH) method to obtain the adjusted *P* values. Log2 fold change (FC) and adjusted *P* values were used to select the differentially expressed mRNAs (DEMs), and the final DEMs, DELs, and DEMis were screened out.

### Function and pathway enrichment analyses of DEMs

Using the enrichment analysis tool DAVID [25] (version 6.8, https://david.ncifcrf.gov/), Gene Ontology (GO) (biological process (BP), cellular component (CC) and molecular function (MF)) analysis was performed, and the results were visualized using GOplot (version 1.0.2) [26]. Kyoto Encyclopedia of Genes and Genomes (KEGG) enrichment analysis was conducted based on the KEGG database [27] using the gene set enrichment analysis (GSEA, version 3.0) [28].

### Co-expression analysis

For DELs and DEMis, the Pearson correlation coefficients between them and with the DEMs were calculated, and correlation tests were performed using the corr.test method in R package psych [29] (ci = F, adjust = "BH"). Multiple test was performed using the BH method. The co-expression pairs were obtained according to the correlation coefficient and significance degree, and the co-expression network was constructed using Cytoscape software (version 3.7.1) [30].

### Online prediction of miRNA

Using starbase (version 3.0, http://starbase.sysu.edu.cn/) [31], the lncRNAs involved in the obtained lncRNA-mRNA co-expression pairs were subjected to lncRNA-miRNA prediction, thus acquiring the lncRNA-miRNA relation pairs. Additionally, the mRNA-miRNA relation pairs were also predicted based on the mRNAs involved in the obtained lncRNA-mRNA co-expression pairs, using the online tool mirwalk (version 3.0) [32]. Moreover, the intersection of these mRNA-miRNA relation pairs with the above mRNA-miRNA co-expression pairs was screened to obtain the final mRNA-miRNA pairs.

### LncRNA and miRNA pathway enrichment analyses

The mRNAs that had co-expression relations with lncRNAs were considered as the target genes of lncRNAs. The lncRNAs with the maximum target genes were subjected to pathway and function enrichment analyses using DAVID [25], and for the lncRNAs with fewer target genes, their functions were searched in genecards (https://www.genecards.org/) [33]. Furthermore, the mRNAs in the mRNA-miRNA pairs were used as the target genes of miRNAs, and the related functions of each miRNA were analyzed in the same way as described above.

### ceRNA relation integration and network construction

From the lncRNA-mRNA pairs with co-expression relations, we selected the lncRNA-mRNA pairs regulated by the same miRNA, which were then integrated with miRNAs to construct the ceRNA network. Cytoscape software (version 3.7.1) [30] was used for network construction.

### Establishment of mRNA prognostic risk model

The mRNAs in the ceRNA network were used as candidate mRNAs, and the univariable Cox regression analysis in R survival package (version 2.44-1.1) [34] was used to analyze the regression coefficient and *P* value of each candidate mRNA in relation to survival time and status. The mRNAs with *P* value < 0.05 were initially considered as the mRNAs related to prognosis.

To further screen the prognostic mRNAs, the calculation model of risk score was defined as follows: Risk score = β gene1*expr (gene 1) + β gene2 * expr (gene 2) + ... + β genen * expr (gene n), Where, β is the prognostic correlation coefficient beta estimated by Cox analysis which equals to log (Hazard Ratio), and expr represents the expression value of corresponding gene.

Thereafter, based on the *P* values of mRNAs in Cox regression analysis ranking in ascending order, the mRNA with the minimum *P* value was used as the starting point, followed by adding the other mRNAs (Table 2). The samples were divided into high-risk and low-risk groups according to the median value of risk core. Survival analysis for high-risk and low-risk groups was performed by log-rank test after the addition of a certain mRNA. Then, the receiver operation characteristic (ROC) curves of 1, 3, and 5 years were calculated using the R survival ROC package (version 1.0.3) [35]. The risk score model with the highest Area Under the Curve (AUC) was taken as the best scoring model.

### Cox regression analysis of prognostic clinical factors

Important clinical factors (gender, age at initial pathologic diagnosis, anatomic neoplasm subdivision, and pathologic stage) were selected from the phenotypic information corresponding to the samples. Thereafter, an univariable Cox regression analysis was performed by combining the survival information, and regression coefficient, and statistical *P* value between each clinical factor, and survival time and state were calculated. The influencing factors with threshold of *P* < 0.05 were further subjected to multivariable Cox regression analysis to obtain the final risk score model.

### Model validation using independent datasets

The prognostic signatures developed were further validated in two independent datasets. The first dataset was the RNA-seq expression profile of colon cancer from the UCSC Xene database. This dataset included HTSeq-FPKM data of 512 samples, clinical phenotype information of 570 samples and survival data of 546 samples. The samples in the training dataset or samples without corresponding survival information or tumor stage information were excluded. At last, 164 samples were included for the validation.

The second dataset was GSE17538 downloaded from Gene Expression Ominibus (GEO), which included 213 colon cancer samples with complete information of survival time and survival status [36, 37].

Risk score was calculated for each individual, and the samples in validation datasets were divided into high-risk group and low-risk group according to the median score of risk score. Kaplan-Meier survival curve analysis was then performed to calculate the difference in the survival prognosis time between the samples of the high and low risk groups.
